# Extrusion Processing of Pure Chokeberry (*Aronia melanocarpa*) Pomace: Impact on Dietary Fiber Profile and Bioactive Compounds

**DOI:** 10.3390/foods10030518

**Published:** 2021-03-02

**Authors:** Vera Schmid, Jan Steck, Esther Mayer-Miebach, Diana Behsnilian, Mirko Bunzel, Heike P. Karbstein, M. Azad Emin

**Affiliations:** 1 Department of Food Technology and Bioprocess Engineering, Max Rubner-Institut (MRI), 76131 Karlsruhe, Germany; vera.schmid@kit.edu (V.S.); diana.behsnilian@mri.bund.de (D.B.); 2 Institute of Process Engineering in Life Sciences, Section I: Food Process Engineering, Karlsruhe Institute of Technology (KIT), 76131 Karlsruhe, Germany; heike.karbstein@kit.edu (H.P.K.); azad.emin@kit.edu (M.A.E.); 3 Institute of Applied Biosciences, Department of Food Chemistry and Phytochemistry, Karlsruhe Institute of Technology (KIT), 76131 Karlsruhe, Germany; jan.steck@kit.edu (J.S.); mirko.bunzel@kit.edu (M.B.)

**Keywords:** arabinan sidechains, monosaccharide composition, glycosidic linkage, pectic arabinan oligosaccharides, dietary fiber stability, polyphenols stability, water solubility index, water absorption index, texture, color

## Abstract

The partial substitution of starch with dietary fiber (DF) in extruded ready-to-eat texturized (RTE) cereals has been suggested as a strategy to reduce the high glycemic index of these food products. Here, we study the impact of extrusion processing on pure chokeberry (*Aronia melanocarpa*) pomace powder (CPP) rich in DF and polyphenols (PP) focusing on the content and profile of the DF fractions, stability of PP, and techno-functional properties of the extrudates. Using a co-rotating twin-screw extruder, different screw speeds were applied to CPP with different water contents (c_w_), which resulted in specific mechanical energies (SME) in the range of 145–222 Whkg^−1^ and material temperatures (T_M_) in the range of 123–155 °C. High molecular weight soluble DF contents slightly increase with increasing thermomechanical stress up to 16.1 ± 0.8 g/100 g dm as compared to CPP (11.5 ± 1.2 g/100 g dm), but total DF (TDF) contents (58.6 ± 0.8 g/100 g dm) did not change. DF structural analysis revealed extrusion-based changes in the portions of pectic polysaccharides (type I rhamnogalacturonan) in the soluble and insoluble DF fractions. Contents of thermolabile anthocyanins decrease linearly with SME and temperature from 1.80 ± 0.09 g/100 g dm in CPP to 0.24 ± 0.06 g/100 g dm (222 Whkg^−1^, 155 °C), but phenolic acids and flavonoids appear to be largely unaffected. Resulting techno-functional (water absorption and water solubility) and physical properties related to the sensory characteristics (expansion, hardness, and color) of pure CPP extrudates support the expectation that granulated CPP extrudates may be a suitable food ingredient rich in DF and PP.

## 1. Introduction

The major component of ready-to-eat texturized (RTE) cereal products, for example, breakfast cereals and snacks, is starch, an easily digestible carbohydrate making these products high glycemic index food. Consumption of high glycemic index food is considered to play an important role in the development of many metabolic disorders, for example, obesity, type 2 diabetes, and cardiovascular diseases [1]. A high intake of dietary fiber (DF) may reduce these risks [2,3,4,5]. Thus, partial or complete substitution of starch with DF-rich materials in RTE cereals is a promising approach towards improving the nutritional quality of these starch-based products [6].

DF is generally classified into insoluble dietary fiber (IDF) and soluble dietary fiber (SDF) based on the water solubility, with SDF being further divided into high molecular weight (HMW-SDF) and low molecular weight (LMW-SDF) SDF. Mainly an increased intake of SDF, present in plant material in lower amounts than IDF, is associated with an improved glycemia and insulin sensitivity in diabetic and non-diabetic persons [2].

Starch plays a fundamental role in expansion and resulting textural characteristics of extruded RTE cereal products [7]. Hence, the effect of starch substitution with DF-rich materials, such as plant derived by-products (pomace), on the techno-functional and sensory characteristics of extruded products is a current research topic [8,9]. The expansion of starch/pomace blends is affected by multiple factors, for example, amount of pomace used and its physical properties, moisture content, and screw speed. Particularly high IDF contents are known to decrease expansion, whereas high SDF contents may result in more expanded extrudates with better overall sensory and techno-functional attributes [7,9,10,11,12]. Simultaneously, extrusion processing may modify the SDF/IDF ratio towards an increase in SDF, by solubilization of the IDF fraction, as already reported for some pomace materials [13,14,15]. This is beneficial considering DF as basic material for extruded products or as starch replacer in RTE cereal products with familiar textural characteristics. Increased SDF contents may also improve the nutritional properties [2].

Chokeberries (*Aronia melanocarpa*) are mainly industrially processed to juice [16], generating considerable amounts of pomace, for example, in Poland up to 10,000 t of chokeberry pomace each year [17]. Though partially used for pectin production or as feed, the large amounts of chokeberry pomace represent an environmental challenge towards sustainable processing [18]. The pomace comprises between 16 and 30% (*w*/*w*) of the unprocessed raw berries [17,19] and is still rich in total dietary fiber (TDF) with about 60 g/100 g dm consisting of about 76% IDF, 20% HMW-SDF, and 4% LMW-SDF [12,20,21] and other bioactive secondary plant metabolites, for example, polyphenols (PP) with a total content (TPP) up to about 5 g/100 g dm [19,20,22]. Several potential health benefits such as cardioprotective and antidiabetic effects have been described [22,23]. The retention of PP from chokeberry extracts, rich in procyanidins, anthocyanins, hydroxycinnamic acids, and flavonols, has been investigated during extrusion cooking using a model based on maize starch [24]. Although partly degraded, a large amount of PP was retained, in particular up to 60% of thermolabile anthocyanins, even at material temperatures (T_M_) of about 180 °C. In another study, chokeberry pomace powder (CPP) was subjected to extrusion-like conditions using a closed cavity rheometer [20]. A shift of the SDF/IDF ratio to increased SDF contents was observed at temperatures ≥140 °C. However, at high temperatures and very long treatment times the SDF was dramatically reduced, whereas the IDF and TDF were increased. The same authors reported that PP were partly degraded due to thermal but not due to mechanical stress. These outcomes are promising with respect to the use of CPP as a potential source of DF and bioactive secondary plant metabolites to partly replace starch in extruded RTE cereals. However, the impact of thermomechanical stress, during extrusion processing in particular, on the DF content and profile of pure CPP has to be studied in detail. 

Thus, the objective of this study was to evaluate the impact of extrusion processing in a twin-screw extruder on pure CPP with a focus on DF stability, DF structures, and PP contents and composition. Furthermore techno-functional (water solubility and water absorption) and physical properties related to the sensory characteristics (sectional and longitudinal expansion, hardness, and color) of extrudates were investigated.

## 2. Materials and Methods

### 2.1. Materials and Reagents

Extrusion trials were performed using commercial chokeberry (*Aronia melanocarpa*) pomace powder (CPP) (Aronia Original Naturprodukte GmbH, Dresden, Germany) [20]. Chemicals and reagents of analytical purity grade were obtained from Merck KGaA (Darmstadt, Germany), unless stated otherwise. Amyloglucosidase (E-AMGDFPD, from *Aspergillus niger*, 36,000 U/g), α-amylase (E-PANAA, from pig pancreas 100,000 U/g), protease (E-BSPRPD from *Bacillus licheniformis* 9000 U/g), Celite 545 and endo-arabinanase (EC 3.2.1.99, from *A. niger*, 9 U/mg) were from Megazyme (Bray, Ireland) and Amberlite FPA53 and Ambersep 200 from Rohm and Haas Europe (Frankfurt, Germany). Thermostable α-amylase (Thermamyl 120 L, EC 3.2.1.1, from *B. licheniformis*, 120 KNU/g), protease (Alcalase 2.5 L, EC 3.4.21.62, from *B. licheniformis*, 2.5 AU/g), and amyloglucosidase (AMG 300 L, EC 3.2.1.3, from *A. niger*, 300 AGU/g) were a gift from Novozymes, (Bagsværd, Denmark). Rotihydroquant C5 and D used for Karl Fischer titration as well as cyanidin-3-O-glucoside (≥96%), cyanidin chloride (≥97%), 5-caffeoylquinic acid (≥97%), quercetin-3-O-glucoside (≥99%), and quercetin dihydrate (≥99%) used as PP standards were obtained from Carl Roth GmbH & Co. KG (Karlsruhe, Germany). Ultrapure water was used for all experiments.

### 2.2. Extrusion Processing

All extrusion trials were carried out using a co-rotating twin-screw extruder (ZSK 26 Mc, Coperion, Stuttgart, Germany) with a barrel length to screw diameter ratio (L/D) of 29 and a screw diameter of 25.5 mm. The extruder barrel consists of seven sections. At the first section, CPP is fed with a constant solid mass flow of 9 and 8 kgh^−1^ using a gravimetrically controlled feeder (DDW-DDSR40, Brabender, Duisburg, Germany). In addition, in the first section, a piston-membrane pump (KM 25, Alldos, Pfinztal, Germany) added water (1 kgh^−1^ and 2 kgh^−1^) to keep the total mass flow constant (10 kgh^−1^). The addition of water resulted in water contents (c_w_) of 13% and 23%, respectively. The extruded material left the extruder by passing a circular die of 3 mm diameter and 10 mm length. All trials were run at screw speeds (n) of 200, 400, 600, and 800 min^−1^ and samples were taken upon achieving steady state conditions. The barrels 2–7 could be heated and cooled separately and temperatures were adjusted to usual values of 40, 60, 80, 100, 100, 100 °C (T_B_ 100 °C). The material temperature (T_M_) was measured at the die entrance using a thermocouple (type J, Ahlborn, Holzkirchen, Germany). Specific mechanical energy (SME) (Whkg^−1^) was calculated by the following Equation (1):

(1) $$\mathrm{SME}=\frac{\frac{n}{n_{\max}}\times \frac{M_{d}-{\;M}_{d,\mathrm{unload}}}{100}}{ṁ}\;\times P_{\max}$$

where n and n_max_ are the actual and maximum screw speed (1800 min^−1^), respectively; M_d_ and M_d,unload_ are the actual and idle torque (%), respectively; ṁ represents the total mass flow (kgh^−1^); and P_max_ represents the maximum engine power (40 kW). All experiments performed are given in Table 1.

Each trial was carried out twice. Immediately after extrusion, the samples were equilibrated at 40 °C for 15 min (Heraeus UT6200, Hanau, Germany), packaged in vacuum bags, stored at −80 °C, and analyzed as described in the following sections. Samples A–D were used to describe the effects of thermomechanical stress. The impact of two different c_w_ at constant n and T_B_ was evaluated comparing A1 and A2 as well as B1/B2, C1/C2, and D1/D2. Samples were also analyzed for dietary fiber (DF) and polyphenols (PP) contents and profiles, as well as for sugar contents in order to evaluate the effects of n and T_B_ at c_w_ 13% (A1, B1, C1, and D1) and of c_w_ at highest n 800 min^−1^ (D1 and D2). 

### 2.3. Dietary Fiber Analysis

#### 2.3.1. Contents of Dietary Fiber Fractions

About 10 g of extruded samples (A1, B1, D1, and D2) were ground and homogenized (mortar and pestle, particle size <500 nm). CPP and all ground extruded samples (two aliquots of about 250 mg, each) were analyzed twice for insoluble DF (IDF), high molecular soluble DF (HMW-SDF), low molecular weight soluble DF (LMW-SDF), and total DF (TDF) contents according to AOAC 2011.25 [25] as modified by McCleary et al. [26]. For LMW-SDF analysis by HPLC-RID (1100 series, Agilent Technologies, Germany) two size exclusion columns in series (Toyo TSKgel, G2500PWXl, 7.8 × 300 mm, Tosoh Bioscience, Griesheim, Germany) were used.

#### 2.3.2. Dietary Fiber Isolation

Preparative isolation of IDF and HMW-SDF fractions of CPP and extruded materials (A1, D1, and D2) was performed, as described earlier [27]. Briefly, mortar ground powder (1 g) was suspended in sodium phosphate buffer and subsequently incubated with thermostable α-amylase, protease, and amyloglucosidase. IDF was separated by centrifugation (4696× *g*, 10 min, Multifuge X1, Thermo Fisher Scientific, Schwerte, Germany) and washed with water, 99.5% ethanol, and acetone. Using the supernatant from previous centrifugation, HMW-SDF fractions were precipitated from 80% ethanol, separated by centrifugation, and washed using 80% aqueous ethanol, 99.5% ethanol, and acetone. Both fiber fractions were vacuum dried at 60 °C (Vacutherm, Thermo Fisher Scientific, Schwerte, Germany) and stored at 20 °C until analysis. In contrast to the method described earlier [27], corrections for residual proteins or ash were not performed.

#### 2.3.3. Monosaccharide Constituents

In order to hydrolyze IDF and HMW-SDF polysaccharides sulfuric acid hydrolysis [28] and methanolysis [29] were carried out, respectively. The monomer composition was determined by high-performance anion-exchange chromatography with pulsed amperometric detection (HPAEC-PAD). The analysis was performed on a Dionex ICS-5000 system equipped with a CarboPac PA-20 column (6.5 µm, 150 × 3 mm, Thermo Fisher Scientific, Schwerte, Germany) at 25 °C. A ternary gradient was applied, as described by Wefers et al. [30] with ultra-pure water (A), 0.1 M sodium hydroxide (B), and 0.1 M sodium hydroxide with 0.2 M sodium acetate (C) at a flow rate of 0.4 mL/min. Results are expressed as mol%.

#### 2.3.4. Monosaccharide Linkage Patterns

Methylation analysis was performed, as described [30,31]. Briefly, IDF and HMW-SDF fractions were swollen in dimethyl sulfoxide, methylated twice using sodium hydroxide and methyl iodide, hydrolyzed using 2 M trifluoroacetic acid, reduced by adding sodium borodeuteride in 2 M aqueous ammonia, and finally acetylated by using methylimidazole as catalyst and acetic anhydride. The partially methylated alditol acetates (PMAA) were analyzed by GC-MS for identification and by GC-FID for relative quantification, using molar response factors described by Sweet et al. [32].

#### 2.3.5. Arabinan Profiling

Pectic arabinan profiling was performed, as previously described by Wefers et al. [33]. Both IDF and HMW-SDF were incubated with endo-arabinanase and liberated oligosaccharides were analyzed by HPAEC-PAD equipped with a CarboPac PA-200 column (5.5 µm, 250 × 3 mm, Thermo Fisher Scientific, Schwerte, Germany) using relative response factors [33]. 

### 2.4. Analysis of Sugar Contents

The sugar contents were analyzed twice in LMW-SDF fractions from CPP and extruded materials (A1, B1, D1, and D2) as prepared according to AOAC 2011.25 [25], as described in Section 2.3.1. Commercial enzyme test kits (R Biopharm AG, Darmstadt, Germany) were used.

### 2.5. Analysis of Polyphenols Contents

Extruded samples (A1, B1, D1, and D2) were homogenized, as described in Section 2.3.1. Two aliquots, each, of CPP and extruded samples (1 g) were suspended in 3% aqueous formic acid (5 mL) and rehydrated for 5 min (ice bath in the dark), and homogenized (Ultra Turrax, IKA-Werke, Germany). PP were extracted five times and four times, respectively, into a mixture of 12% water, 3% formic acid, and 85% methanol (*v*/*v*/*v*) and anthocyanins, phenolic acids, and flavonols were measured by HPLC-DAD (1100 series, Agilent Technologies, Germany), as described earlier [18]. Total polyphenols contents (TPP) were determined using the unspecific Folin–Ciocalteu test and quantified using catechin monohydrate as standard compound [34].

### 2.6. Residual Moisture Content

Karl Fischer titration (Titroline alfa, Schott Instruments GmbH, Mainz, Germany) was used to determine the residual moisture content of CPP and extruded samples (A1, B1, D1, and D2) six times each [35].

### 2.7. Analysis of Techno-Functional and Sensory Relevant Physical Properties 

#### 2.7.1. Water Absorption Index, Water Solubility Index 

CPP as well as all extruded samples were milled using a coffee mill (M55, Petra Electric, Ense, Germany), and then sieved to particle size between 0.07 and 0.14 mm. Sieved powders were dried in a vacuum dryer (VT 5042 EK, Heraeus, Hanau, Germany) at 40 °C and 8 mbar. The water solubility index (WSI) and water absorption index (WAI) were determined according to Anderson [36] with slight modifications. Sieved powders (0.5 g) were added to 19.5 g of demineralized water, and the suspensions were mixed for 1 min (REAX top, Heidolph Instruments GmbH & Co. KG, Schwabach, Germany). The samples were shaken at 200 min^−1^ (Orbital shaker Incubator SI 50, Stuart, Staffordshire, United Kingdom) for 24 h at room temperature (about 25 °C) and centrifuged at 4600× *g* for 50 min at 20 °C (Rotanta 460 R, Andreas Hettich GmbH & Co. KG, Tuttlingen, Germany). After separation, the wet precipitates were weighed directly, whereas the supernatants were dried for 72 h at 80 °C (T-6060 Heraeus, Hanau, Germany) before weighing. WAI and WSI were calculated according to the following Equations (2) and (3):

(2) $$\mathrm{WAI}=\;\frac{m_{\mathrm{water}}\;-{\;m}_{\mathrm{wet}\;\mathrm{supernatant}}}{m_{\mathrm{initial}\;\mathrm{sample}\;\mathrm{weight}}}$$

(3) $$\mathrm{WSI}=\;\frac{m_{\mathrm{dried}\;\mathrm{supernatant}}}{m_{\mathrm{initial}\;\mathrm{sample}\;\mathrm{weight}}}$$

Analyses were performed in triplicate.

#### 2.7.2. Expansion Indices

The sectional expansion index (SEI) and longitudinal expansion index (LEI) are parameters to describe the expansion of extruded products. The SEI is defined as the ratio of the cross-sections of the expanded extrudate and the die (Equation (4)) as follows:

(4) $$\mathrm{SEI}=\;{\left(\frac{d_{\mathrm{ext}}}{d_{\mathrm{die}}}\right)}^{2}$$

The die diameter d_die_ was 3 mm for all extrusion trials. The diameter of extrudates d_ext_ was determined 6 times by using a caliper 24 h after extrusion.

The LEI is defined as ratio of the extrudate velocity v_ext_ after the die exit and the melt velocity inside the die v_die_. Extrudate samples were taken manually for a period of 3 s and the final lengths were measured. The LEI was calculated according to Equation (5) using Equations (6) and (7) as follows:

(5) $$\mathrm{LEI}=\;\frac{v_{\mathrm{ext}}}{v_{\mathrm{die}}}$$

(6) $$v_{\mathrm{ext}}=\;\frac{l}{t}$$

(7) $$v_{\mathrm{die}}=\;\frac{ṁ}{A_{\mathrm{die}}{\times \;\rho }_{\mathrm{die}}}$$

where l is the measured length of the extrudate, t the time for sampling (3 s), ṁ the total mass flow (10 kgh^−1^), A_die_ the area of the die (7.07 m^2^), and ρ_die_ the density of matrix (1400 kgm^−3^) [37].

#### 2.7.3. Hardness

Hardness of the product was measured by using a texture analyzer (Z2.5 TS, ZwickRoell, Ulm, Germany). A Kramer shear cell with one blade was used for testing (settings of pretest speed of 0.1 mmin^−1^, test speed of 0.01 mmin^−1^, test distance 6 mm, and pre-force 0.2 N). Hardness was determined four times. 

#### 2.7.4. Color

The color of the sieved and dried, samples was measured by a spectral photometer (CM 700d, Konica Minolta, Marunouchi, Tokyo, Japan). The powder was placed on a white sheet. The color was determined three times.

### 2.8. Statistics

Structural data of isolated DF (monosaccharide constituents and linkage patterns, arabinan profiling) are reported as mean ± range/2; all other data are presented as mean ± standard deviation (SD). ANOVA followed by Holm–Sidak test was performed to determine statistical significance (*p* < 0.05) between groups using SigmaPlot software (version 13.0, Systat Software GmbH, Erkrath, Germany). Pearson’s correlation coefficient (r and *p* values) were reported to describe the linear correlation between techno-functional and sensory relevant physical properties and SME or T_M_, respectively.

## 3. Results and Discussion

### 3.1. Extrusion Processing

**Figure 1:**
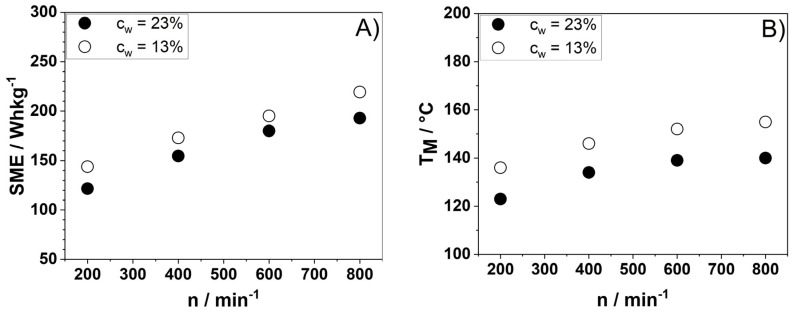
Effect of screw speed (n) and water content (c_w_) on (**A**) specific mechanical energy (SME) and (**B**) the chokeberry pomace powder (CPP) material temperature (T_M_) at 100 °C barrel temperature (T_B_).

Specific mechanical energy (SME) and material temperature (T_M_) give information on the thermomechanical stress profiles achieved during extrusion processing and are commonly used to evaluate the impact of processing on nutritional and techno-functional attributes of extrudates. As expected, an increase in screw speed (n) from 200 to 800 min^−1^ led to an increase in SME by 35% for a water content (c_w_) of 13% (Figure 1A). Increasing the c_w_ to 23% (total mass flow was kept constant at 10 kgh^−1^) at different *n* results in decreases in SME by 8 to 18% (Table 1) due to a reduction in the matrix viscosity and subsequently lower shear stresses [38]. Figure 1B shows the T_M_ at the die entrance, shortly before exiting the extruder, for both c_w_ 13% and 23%. The lowest T_M_ of 123 °C is measured at c_w_ 23% and the lowest n of 200 min^−1^. Increasing n and reducing c_w_ increases T_M_ up to 155 °C. T_M_ was always significantly higher than T_B_, which was kept constant at 100 °C, indicating a high viscous dissipation energy resulting from mechanical stresses.

### 3.2. Thermomechanical Stability of Dietary Fiber

Data about the impact of extrusion processing on the dietary fiber (DF) structure of pure fruit pomaces are scarce. However, both *Aronia melanocarpa* and *Malus domestica* (apple) bear the same botanical fruit type (pome) and both are members of the Rosaceae subtribe Malinae. Therefore, the results are compared to extruded pure apple pomace wherever possible.

#### 3.2.1. Dietary Fiber Contents

Individual DF (insoluble DF, IDF; high molecular weight soluble DF, HMW-SDF; and low molecular weight soluble DF, LMW-SDF) as well as total DF (TDF) contents of chokeberry pomace powder (CPP) before and after extrusion processing are shown in Table 2. 

The contents of TDF and LMW-SDF remain unchanged irrespective of the SME applied and the materials c_w_. Likewise, no significant changes in the contents of IDF, the main fiber fraction, are identified. Yet, at the highest SME (D1, 222 ± 10 Whkg^−1^) the IDF content tends to be slightly reduced by about 5%. In contrast, at the highest SME (D1), the HMW-SDF contents are enhanced with a significant increase of about 40%. This reflects the solubilization of proportions of IDF arabinans which results in higher amounts of soluble arabinans in HMW-SDF (see below). The SDF/IDF ratio is shifted from 0.32:1 in CPP to 0.42:1 under D1 conditions. An increasing solubilization of DF with increasing SME was also reported for the extrusion of pure apple pomace [39]. A higher c_w_ (D2, 23%) reduces SME and T_M_ (Table 2) but has no impact on the HMW-SDF content as compared with extrusion at lower c_w_ (D1, 13%). Different from the results of this study, the IDF contents increased largely during defined thermomechanical treatment in a closed cavity rheometer after long-time treatment (20 min) at 140 °C and 160 °C (shear rate 50 s^−1^, c_w_ 12%) [20]. The nearly unmodified IDF contents after extrusion at all applied conditions are explained by the very short residence time of the material within the extruder, in the range of seconds, which is one of the advantages of short-time extrusion at high temperatures [40,41]. 

The knowledge gained here about the impact of extrusion processing of pure chokeberry pomace on the structure and contents of its DF is new, as literature data on the extrusion of pure fruit pomace is scarce. Many papers describe the impact of extrusion on the DF of fruit pomace and starch blends. However, in such studies it is difficult to distinguish between the impact of extrusion processing on starch and on the non-starch polysaccharides from pomace. In this study, the extrusion of pure CPP was investigated focused on the possible impact of processing on the content and profile of the HMW-SDF fraction [13,14,15] which is associated with beneficial health-related effects [2]. Extrusion results in an increase in this DF fraction and extrudates contain up to 60% of TDF. These results suggest that, with regard to possible nutritional benefits, pure CCP extrudates could be used as DF-rich food ingredients for fiber enrichment. Furthermore, it was of scientific interest to validate our previous data on the effects of thermal and mechanical stress investigated using a closed cavity rheometer [20].

#### 3.2.2. Changes in Monosaccharide Composition

**Figure 2:**
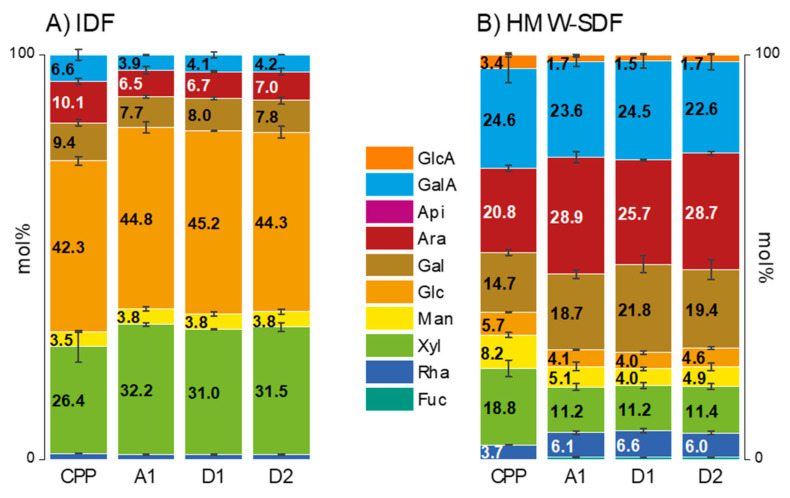
Monosaccharide composition of (**A**) insoluble dietary fiber (IDF) and (**B**) high molecular weight soluble dietary fiber (HMW-SDF) from chokeberry pomace powder (CPP) and samples after extrusion processing at different water contents (c_w_) and specific mechnical energies (SME) applied resulting in different material temperatures (T_M_) (mol%, range/2, *n* = 2). (A1) SME 145 ± 6 Whkg^−1^, T_M_ 136 °C; (D1) SME 222 ± 10 Whkg^−1^, T_M_ 155 °C; (D2) SME 190 ± 9 Whkg^−1^, T_M_ 140 °C. GlcA, d-glucuronic acid; GalA, d-galacturonic acid; Api, d-apiose; Ara, l-arabinose; Gal, d-galactose; Glc, d-glucose; Man, d-mannose; Rha, l-rhamnose; Xyl, d-xylose; Fuc, l-fucose.

Extrusion processing causes only minor modifications in the monosaccharide composition of the IDF fraction of CPP (Figure 2A). 

As shown earlier [20], the monosaccharide composition of IDF from CPP consists mostly of glucose, the majority originating from cellulose and xylose, mostly originating from hemicelluloses. Less dominant monomers such as arabinose, galactose, and galacturonic acid originate from insoluble pectic polysaccharides. Only the portions of monosaccharides from insoluble pectins decrease slightly from 26.1 mol% to 18.1–19.0 mol% in total during extrusion irrespective of c_w_ and SME applied. The monosaccharide composition of HMW-SDF from CPP shows more distinct modifications after extrusion processing (Figure 2B). Arabinose, galactose, and rhamnose portions that can be assigned to rhamnogalacturonan type I with its specific sidechains (arabinans and galactans, see also Section 3.2.3) rise markedly from 39.2 mol% to 53.7–54.1 mol% in total. Again, these effects seem to be independent of SME and c_w_. Overall, due to thermomechanical processing, ratios of pectic sidechain monomers decrease in the IDF, whereas they increase in the HMW-SDF, indicating a solubilization of pectins rather than a degradation of pectic polysaccharides from IDF. However, both processes, solubilization of polysaccharides and degradation of specific structural elements of these polysaccharides, are likely to occur to some extent concurrently. Similar findings were obtained by Hwang et al. [39] who described that extrusion processing of apple pomace reduced arabinose and galactose ratios in insoluble polysaccharide fractions. The authors assumed that extrusion cleaves preferentially arabinogalactan sidechains from pectins [39]. Unfortunately, no additional data on the neutral sugar composition of soluble polysaccharide fractions or polysaccharide linkage types of extrusion processed apple pomace polysaccharides were presented. 

#### 3.2.3. Polysaccharide Linkage Types and Ratios of Extruded Materials 

Polysaccharide linkage types and ratios of CPP before and after extrusion processing are shown in Table 3. Overall, the main components of IDF are 1,4-glucopyranose (40.4–48.6 mol%) originating primarily from cellulose, various arabinofuranose units (14.2–25.8 mol% in total) primarily from arabinan sidechains, and 1,4-xylopyranose (8.4–11.9 mol%) originating from xylans. These findings are in accordance with the assignments based on the monosaccharide compositions. Extrusion processing causes distinct alterations especially on arabinan monomers such as terminal, 1,5-, 1,2,5-, 1,3,5- and 1,2,3,5-linked arabinofuranose units. In fact, the total portion of these five structural elements of IDF pectic polymers decreases with increasing SME and T_M_ from 22.9 mol% in CPP to 15.0 mol% (A1, SME 145 ± 6 Whkg^−1^, 136 °C) and 11.7 mol% (D1, SME 222 ± 10 Whkg^−1^, 155 °C), respectively. With an increase in c_w_ from 13% to 23% leading to lower SME and T_M_ (D2, SME 190 ± 9 Whkg^−1^, 140 °C), the portion of these pectic monomers (17.7 mol% in total) was less reduced as compared with extrusion at lower c_w_ (D1). At the same time, ratios of 1,4-glucopyranose increase from 40.4 mol% (CPP) to 47.6 mol% (A1), 48.6 mol% (D1), and 42.6 mol% (D2), respectively. Furthermore, aforementioned arabinan elements strongly increase in HMW-SDF due to extrusion processing. Initially, summed up arabinofuranose units account for 37.7 mol% in untreated CPP, but increase to 56.1 mol% (A1), and 51.2 mol% (D1) (both c_w_ 13%), and 57.3 mol% (D2, c_w_ 23%) in extruded materials. Portions of 1,2-rhamnopyranose, 1,2,4-rhamnopyranose, and 1,4-galactopyranose also increase, supporting our assumption that extrusion affects primarily rhamnogalacturonan type I including its neutral sidechains such as arabinans, respectively. Thus, data from methylation analysis also support the idea that solubilization of insoluble pectins appears to be more important under the conditions used than degradation of specific structural units from IDF polysaccharides due to thermomechanical treatment.

#### 3.2.4. Arabinan Structural Details

**Figure 3:**
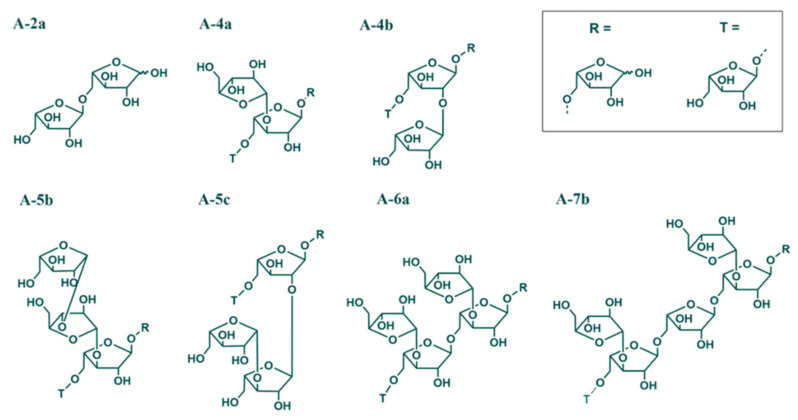
Pectic arabinan oligosaccharides liberated by endo-arabinanase from dietary fiber fractions isolated from chokeberry pomace powder (CPP) before and after extrusion processing. T, terminal end; R, reducing end [20].

Our prior work [20] showed that chokeberry pomace DF arabinans are largely based on the structural elements A-2a, A-5b, and A-4a oligosaccharides, as shown in Table 4 and Figure 3. These structures represent linear and O3-branched arabinose units, whereas A-4b and A-5c, the latter exclusively in IDF, contain O2-branched arabinose units. A-6a and A-7b demonstrate more highly branched arabinans structures [33]. Extrusion processing does not result in major changes in HMW-SDF. The IDF arabinan motives show some minor modifications. The A-5c and A-4b motives are no longer detectable after extrusion processing almost irrespective of SME applied. However, in general, data from the arabinan profiling approach indicate that pectic polymer solubilization and modifications are not a consequence of overall specific structural changes with the exception, that O2 branches appear to be slightly less stable than O3 branches. Whether or not this hypothesis is correct needs to be clarified in future studies.

### 3.3. Sugar Contents

Increasing the SME (A1 < B1 < D2 < D1) reduces initial glucose and fructose contents of CPP by about 15–25% and about 13–27%, respectively (Table 5). This indicates the possible formation of Maillard glycation products, as previously suggested [42]. Sensory properties of extruded products are based to some extend on these compounds [43,44]. Some authors also ascribed beneficial health effects of Maillard products [45]. Sucrose contents remain unchanged during extrusion processing. The data presented are in accordance with data on thermal and mechanical treatment in a closed cavity rheometer, as published earlier [20].

### 3.4. Stability of Polyphenols

**Figure 4:**
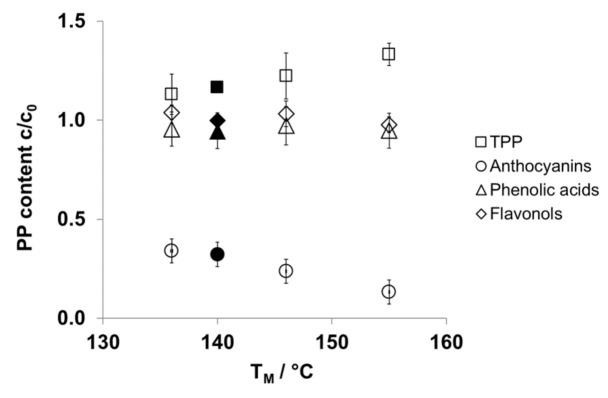
Thermomechanical impact on polyphenol (PP) stability in chokeberry pomace powder (CPP) after extrusion processing (mean ± SD, *n* = 2). c_0_, based on CPP; T_M_, material temperature. Open symbols, water content (c_w_) 13% (A1, B1, D1). Filled symbols, c_w_ 23% (D2). TPP, total PP.

As expected after thermal processing [46], the total content of monomeric anthocyanins in CPP (1.80 ± 0.09 g/100 g dm) decreases by about 65% due to extrusion processing at a T_M_ of 136 °C ((A1) c_w_ 13%, SME 145 ± 6 Whkg^−1^) and is linearly reduced with increasing SME and T_M_ (B1 and D1). A residual content of 0.24 ± 0.06 g/100 g dm is still detected after the highest SME applied (Figure 4 and Table 6). 

Anthocyanin degradation may be reduced by adjustment of the water dosage, for example, about 19% more of the initial anthocyanins are retained by raising c_w_ from 13% (D1) to 23% (D2). This is in accordance with literature data describing the effect of extrusion processing of berry materials, for example, blueberry, cranberry, and raspberry, used as fruit powders, pomace, or combined at different ratios with starch. Other authors reported total anthocyanin losses between 30% and about 90% depending on T_B_, SME, and c_w_ [47,48,49]. Retention of cyanidin glycosides of about 17% and 58% have also been described for a chokeberry extract extruded in a starchy matrix at c_w_ 15% and 22% (SME 219 ± 2 and 154 ± 13 Whkg^−1^), respectively [24]. The effects, degradation and retention of total anthocyanins, were also both found for individual cyanidin glycosides (Figure 4 and Table 6).

Independent of the SME applied, the aglycon (cyanidin) is generated during processing indicating deglycosylation as the first step in the degradation of chokeberry anthocyanins [46,50]. 

While anthocyanins are degraded, both phenolic acids and flavonols are almost entirely retained (95 ± 9%, 97 ± 10% and 104 ± 4%, 103 ± 6%, respectively) during extrusion processing up to T_M_ 146 °C (A1 and B1) (Figure 4 and Table 7). 

Moreover, total phenolic acids contents remain more or less unchanged at the highest T_M_ (155 °C, D1), while increasing c_w_ (D2) does not show a protective effect. However, individual phenolic acid isomers show different stability. Whereas 5- and 3-caffeoylquinic acid are entirely retained, the content of the very minor isomer 4-caffeoylquinic increases with increasing SME and T_M_ at low c_w_ (increase of 82% in D1), probably due to isomerization during thermal processing [51], but practically no isomerization is observed increasing c_w_ (D2). The contents of total flavonols, as well as of the aglycon quercetin and all glycosides (quercetin-3O-vicianoside, quercetin-3O-robinobioside, quercetin-3O-galactoside, quercetin-3O-rutinoside, quercetin-3O-glucoside), remain unchanged. This is in contrast to the findings of other authors [49], who identified 30–34% higher flavonol contents of all extrudates as compared with the unextruded cranberry pomace. They explained the increase by an enhanced extraction of the compounds bound to cell wall components as a result of thermal matrix disruption.

Finally, extrusion processing reduces the content of polyphenols (sum of monomeric anthocyanins, phenolic acids, and flavonols, TPP-HPLC) to about 31% to 48% at the different SME applied, whereas the measured total polyphenols (TPP) content, in contrast, is enhanced to 113–133% (Table 8). 

It has to be kept in mind that the Folin–Ciocalteu assay is not specific only for phenolic compounds [34], but other reducing compounds present in the sample, for example, reducing compounds resulting from the Maillard reaction are recorded at the same time [8,49]. Moreover, increased TPP contents may also indicate a higher extractability of proanthocyanidins, which have been described as the main class of TPP in chokeberry pomace [17,23]. Oligo- and polymeric proanthocyanidins have been shown to adsorb to apple cell-wall polysaccharides via hydrogen bonds with a high affinity in particular to pectic polysaccharides, in particular arabinans, and high T_M_ during thermomechanical treatment may enhance their extractability [52,53]. Overall, these findings confirm the results of our previous study on the stability of polyphenols when applying defined thermal and mechanical stress in a closed cavity rheometer [20]. Combined with known health-related properties of chokeberry polyphenols [22], these data suggest that chokeberry pomace powder is a valuable source of polyphenols for the fortification of extruded food. 

### 3.5. Techno-Functional and Sensory Relevant Physical Properties

The effect of extrusion processing on techno-functional (water solubility and water absorption) and physical properties related to the sensory characteristics (sectional and longitudinal expansion, hardness, and color) is shown in Table 9. 

#### 3.5.1. Hydration Properties

Hydration properties can be described by the water solubility index (WSI) and the water absorption index (WAI). The WSI describes the amount of material that is dissolved in water, whereas the WAI describes the amount of water absorbed by the material. Depending on the extrusion conditions, hydration properties can be modified. Thus, these values can be used to describe the extent to which the fiber structure (on a macroscopic and/or molecular level) was modified by extrusion processing. The WAI of untreated CPP (2.92 ± 0.08) is comparable to blueberry pomace (2.66 ± 0.05) but lower than apple pomace (5.60 ± 0.08) and higher than starch (1.15 ± 0.09) [9]. Through extrusion, the WAI is reduced to about 2 without an impact of n and c_w_ applied (Table 9). No linear correlation between WAI and SME or T_M_ were found. The WSI of CPP extruded at SME 145 ± 6 Whkg^−1^ and c_w_ 13% (A1) is in the range of the untreated CPP and increases almost linearly (r = 0.88, *p* = 0.004) with increasing SME by about 4% at the highest SME (D1) (Table 9). There is very little literature dealing with the extrusion of unblended pomace in a twin-screw extruder. For the extrusion of apple pomace, which is also a member of the Rosaceae plant family, similar results have been reported. The WSI value of raw apple pomace was in the same range as CPP and increased with increasing thermomechanical treatment (SME, T_M_). However, the increase was more pronounced than for chokeberry [54]. The increase in the WSI is in accordance with increased HMW-SDF contents detected (Table 2). However, it has to be kept in mind that sugar contents are reduced during processing (Table 5). Enhanced c_w_ during processing (D2, 23%) at SME 190 Whkg^−1^ slightly reduces the WSI.

#### 3.5.2. Expansion

The expansion of extruded cereal products is described by sectional and longitudinal expansion indices, SEI and LEI, respectively. SEI values between 0.85 and 1.95 are measured for all CPP extrusion trials. A slight reduction is observed with increasing n, SME (r = −0.83, *p* = 0.010) and T_M_ (r = −0.74, *p* = 0.034). Mostly, additional water increases SEI, except for A2. In contrast, SEI values between 1 and 15 are to be expected for pure starch [55], whereas SEI values of starch-based materials typically range between 10 and 20 [56]. The LEI increases with n as expected. Furthermore, an almost linear correlation to SME (r = 0.92, *p* = 0.001) was observed. However, T_M_ (r = 0.80, *p* = 0.016) was less correlated with the LEI values. Increasing c_w_ resulted in slightly lower LEI. As described above (Figure 2), CPP composition and structure change slightly with varying process parameter. Therefore, no large changes in expansion based on structural changes were expected and the slight changes in expansion observed are explained by the influence of process parameters. 

#### 3.5.3. Hardness

**Figure 5:**
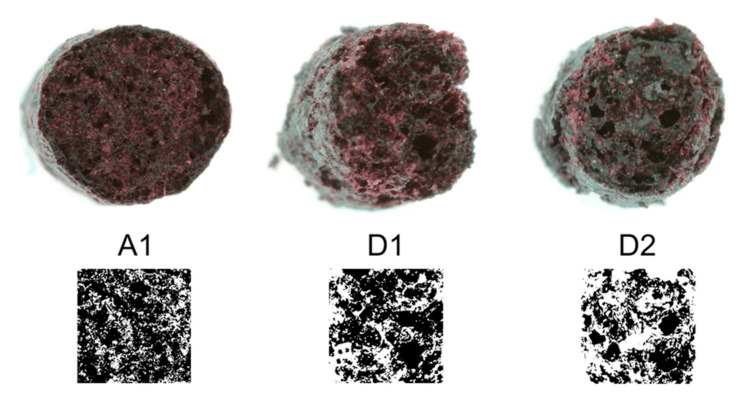
Cross section and porosity of chokeberry pomace powder (CPP) extrudates after extrusion processing at different specific mechanical energies (SME) applied at different water contents (c_w_). (A1) SME 145 ± 6 Whkg^−1^, c_w_ 13%; (D1) SME 222 ± 10 Whkg^−1^, c_w_ 13%; (D2) SME 190 ± 9 Whkg^−1^, c_w_ 23%.

The extruded samples are similar in hardness (Table 9). There is only a slight reduction in hardness with increasing n from 200 to 800 min^−1^ observed for both water contents (A1, B1, C1, D1 and A2, B2, C2, D2). Briefly, the higher the SME and T_M_, the softer the extrudates ((SME) r = −0.81, *p* = 0.014 and (T_M_) r = −0.82, *p* = 0.012). In general, CPP extrudates are much softer than common RTE cereals consisting of starch-based materials with a typical hardness of about 25 Nm^−2^ [57]. Due to comparable hardness and longitudinal, as well as sectional expansion, no change in porosity is observed (Figure 5).

#### 3.5.4. Color

Compared to CPP, the extruded material had significantly lower L*, a*, and b* values (Table 9). Thus, extruded products are, in general, darker (lower L* value) and less reddish (lower a* value) possibly due to the formation of colored Maillard reaction products [20]. Irrespective of various extrusion conditions (A1, A2, B1, B2, C1, C2, D1, and D2), the L* values (21.7–23.5), the a* values (16.0–17.5), and the b* values (1.5-1.9) do not differ significantly (Table 9) and could not be correlated linearly to SME or T_M_ decrease despite the decrease in coloring anthocyanins (Table 6). 

## 4. Conclusions

No significant changes in total DF contents occur during extrusion processing of pure CPP in a co-rotating twin-screw extruder applying SME in the range of 145–222 Whk^−1^, typical for the production of RTE cereals. Although the composition of the individual DF fractions (IDF and HMW-SDF) is modified during extrusion, we assume that these modifications are mostly based on solubilization of the pectic rhamnogalacturonan type I polymers; degradation of specific structural elements of cell wall polysaccharides seems to be less pronounced. Sugar contents are reduced with increasing SME. Anthocyanin contents decrease linearly with SME, whereas contents of phenolic acids and flavonols remain unaffected. On the basis of these data, after extrusion processing CPP remains a valuable source of both, DF and PP, and has the potential to partially substitute starch in RTE cereals, for example, crispy breakfast cereals and snacks. As expected, the techno-functional and sensory relevant physical properties of pure CPP extrudates, analyzed and presented here, are different as compared with common starch-based RTE cereals. However, due to a moderate crispness and hardness, as well as an appealing color, together with a surprisingly overall appealing visual impression and pleasant taste (as evaluated by the working group and lab colleagues), granulated CPP extrudates may potentially be used as a food ingredient for DF and PP enrichment after further sensory analysis confirmed the assumption. 

## Figures and Tables

**Table 1:** Experiments performed. Extrusion parameters (n, screw speed (min^−1^); T_B_, barrel temperature (°C); c_w_, water content (%); SME, specific mechanical energy (Whkg^−1^); T_M_, material temperature (°C); total mass flow for all trials was 10 kgh^−1^).

Sample Name	n	T_B_	c_w_	SME	T_M_
A1	200	100	13	145 ± 6	136
A2	200	100	23	122 ± 5	123
B1	400	100	13	174 ± 4	146
B2	400	100	23	155 ± 3	134
C1	600	100	13	195 ± 10	152
C2	600	100	23	180 ± 7	139
D1	800	100	13	222 ± 10	155
D2	800	100	23	190 ± 9	140

**Table 2:** Dietary fiber (DF) contents of chokeberry pomace powder (CPP) before and after extrusion processing (g/100 g dm, mean ± SD, *n* = 2) at water contents of 13% (A1, B1, and D1) and 23% (D2), respectively. SME, specific mechanical energy (Whkg^−1^); T_M_, material temperature (°C); IDF, insoluble DF; HMW-SDF, high molecular weight soluble DF; LMW-SDF, low molecular weight soluble DF; TDF, total DF.

Sample	SME	T_M_	IDF	HMW-SDF	LMW-SDF	TDF
CPP	---	---	43.8 ± 1.4 ^A^	11.5 ± 1.2 ^A^	2.4 ± 2.2 ^A^	57.8 ± 2.0 ^A^
A1	145 ± 6	136	44.3 ± 1.1 ^A^	14.5 ± 0.9 ^AB^	0.8 ± 0.4 ^A^	59.6 ± 0.7 ^A^
B1	174 ± 4	146	42.9 ± 1.8 ^A^	13.9 ± 0.7 ^AB^	1.1 ± 0.3 ^A^	58.0 ± 2.3 ^A^
D1	222 ± 10	155	41.5 ± 2.8 ^A^	16.1 ± 0.8 ^B^	1.5 ± 0.1 ^A^	59.0 ± 1.8 ^A^
D2	190 ± 9	140	43.3 ± 2.2 ^A^	14.3 ± 1.2 ^AB^	1.1 ± 0.4 ^A^	58.7 ± 3.8 ^A^

Means with different superscript capital letters within the same column differ significantly (*p* < 0.05).

**Table 3:** Partially methylated alditol acetates (PMAA) from insoluble dietary fiber (IDF) and high molecular weight soluble dietary fiber (HMW-SDF) fractions isolated from chokeberry pomace powder (CPP) before and after extrusion processing ^1^ (mol%, range/2, *n* = 2).

	IDF				HMW-SDF			
Glycosidic Linkage ^2^	CPP	A1	D1	D2	CPP	A1	D1	D2
t-Ara*f*	7.0 ± 0.6	5.2 ± 0.7	3.8 ± 0.5	4.9 ± 1.0	15.7 ± 0.9	18.5 ± 0.6	16.9 ± 2.4	18.6 ± 0.2
t-Ara*p*	0.9 ± 0.1	0.8 ± 0.1	0.9 ± 0.1	1.1 ± 0.2	1.9 ± 0.1	1.2 ± 0.4	1.5 ± 0.1	1.5 ± 0.4
1,2-Ara*f*	0.3 ± 0.1	0.5 ± 0.2	0.5 ± 0.1	0.8 ± 0.2	0.9 ± 0.3	0.6 ± 0.3	0.5 ± 0.1	0.6 ± 0.2
1,3-Ara*f*	1.8 ± 0.3	1.5 ± 0.1	1.2 ± 0.1	2.0 ± 0.4	3.3 ± 0.4	4.4 ± 0.5	4.1 ± 0.1	4.2 ± 0.3
1,5-Ara*f*	10.4 ± 1.3	6.9 ± 0.2	5.9 ± 1.9	9.7 ± 4.2	17.0 ± 1.0	27.6 ± 0.3	24.9 ± 4.2	28.2 ± 0.8
1,2,5-Ara*f*	0.8 ± 0.4	0.2 ± 0.1	0.2 ± 0.1	0.3 ± 0.1	0.7 ± 0.1	0.9 ± 0.1	0.9 ± 0.1	0.9 ± 0.1
1,3,5-Ara*f*	3.9 ± 0.2	2.2 ± 0.1	1.4 ± 0.1	2.0 ± 0.2	3.1 ± 0.2	7.8 ± 0.3	7.1 ± 1.5	8.2 ± 0.1
1,2,3,5-Ara*f*	0.8 ± 0.1	0.5 ± 0.1	0.4 ± 0.1	0.8 ± 0.1	1.2 ± 0.3	1.3 ± 0.1	1.4 ± 0.2	1.4 ± 0.2
Σ Ara	25.8 ± 1.7	17.8 ± 0.8	14.2 ± 2.7	21.6 ± 6.0	43.8 ± 2.3	62.3 ± 0.1	57.3 ± 8.4	63.5 ± 1.3
t-Gal*p*	3.1 ± 0.3	3.3 ± 0.1	3.1 ± 0.1	3.5 ± 0.4	4.7 ± 0.5	4.6 ± 0.2	4.7 ± 0.6	4.5 ± 0.2
1,2-Gal*p*	ND	ND	ND	ND	1.2 ± 0.3	0.1 ± 0.1	0.5 ± 0.2	0.1 ± 0.1
1,3-Gal*p*	0.2 ± 0.1	0.2 ± 0.1	0.1 ± 0.1	0.2 ± 0.1	1.8 ± 0.1	0.9 ± 0.3	1.0 ± 0.2	0.9 ± 0.3
1,4-Gal*p*	2.9 ± 0.4	3.3 ± 0.2	2.7 ± 0.2	3.3 ± 0.8	1.4 ± 0.6	4.3 ± 1.0	6.9 ± 1.6	4.2 ± 0.7
1,6-Gal*p*	0.7 ± 0.1	0.8 ± 0.1	0.8 ± 0.1	0.8 ± 0.1	2.2 ± 0.2	1.2 ± 0.2	1.3 ± 0.2	1.1 ± 0.1
1,3,4-Gal*p*	0.2 ± 0.1	0.2 ± 0.1	0.2 ± 0.1	0.4 ± 0.1	ND	ND	ND	ND
1,3,6-Gal*p*	0.7 ± 0.1	0.4 ± 0.1	0.4 ± 0.1	0.5 ± 0.2	6.5 ± 0.8	3.2 ± 0.7	3.0 ± 0.4	2.6 ± 0.1
Σ Gal	7.8 ± 0.7	8.2 ± 0.2	7.4 ± 0.1	8.9 ± 0.7	17.8 ± 1.0	14.4 ± 2.3	17.4 ± 3.1	13.6 ± 0.9
t-Glc*p*	1.1 ± 0.3	1.2 ± 0.1	1.4 ± 0.3	1.1 ± 0.2	1.7 ± 0.6	0.9 ± 0.3	0.6 ± 0.1	0.9 ± 0.2
1,4-Glc*p*	40.4 ± 0.1	47.6 ± 0.7	48.6 ± 3.9	42.6 ± 4.5	8.8 ± 1.0	3.6 ± 0.9	5.7 ± 3.4	3.4 ± 0.9
1,4,6-Glc*p*	3.7 ± 0.4	4.0 ± 0.1	4.0 ± 0.1	3.8 ± 0.5	2.8 ± 0.6	1.9 ± 0.1	2.6 ± 0.5	1.9 ± 0.7
Σ Glc	45.2 ± 0.2	52.8 ± 0.8	54.1 ± 4.2	47.4 ± 4.2	13.3 ± 2.1	6.4 ± 1.2	9.0 ± 4.0	6.2 ± 1.8
t-Man*p*	0.2 ± 0.1	0.2 ± 0.1	0.3 ± 0.1	0.2 ± 0.1	2.4 ± 1.3	0.7 ± 0.2	0.4 ± 0.1	0.6 ± 0.2
1,4-Man*p*	2.6 ± 0.4	3.3 ± 0.3	3.4 ± 0.1	3.2 ± 0.2	2.2 ± 0.4	1.6 ± 0.2	1.7 ± 0.1	2.0 ± 0.6
1,4,6-Man*p*	0.5 ± 0.1	0.6 ± 0.1	0.7 ± 0.1	0.6 ± 0.1	0.8 ± 0.2	ND	ND	ND
Σ Man	3.3 ± 0.4	4.2 ± 0.3	4.3 ± 0.1	4.1 ± 0.3	5.4 ± 1.8	2.2 ± 0.3	2.2 ± 0.2	2.7 ± 0.8
1,2-Rha*p*	0.6 ± 0.3	0.5 ± 0.1	0.3 ± 0.1	0.4 ± 0.2	0.8 ± 0.1	1.7 ± 0.1	1.6 ± 0.3	1.6 ± 0.1
1,2,4-Rha*p*	0.3 ± 0.1	0.6 ± 0.1	0.5 ± 0.1	0.6 ± 0.2	0.4 ± 0.1	0.9 ± 0.1	1.1 ± 0.1	0.8 ± 0.1
Σ Rha	0.9 ± 0.4	1.1 ± 0.1	0.8 ± 0.2	1.0 ± 0.1	1.2 ± 0.1	2.7 ± 0.1	2.6 ± 0.4	2.4 ± 0.1
t-Xyl*p*	5.0 ± 0.1	5.2 ± 0.4	4.7 ± 0.1	5.4 ± 0.3	4.3 ± 0.5	4.7 ± 0.4	4.9 ± 0.2	5.1 ± 0.2
1,2-Xyl*p*^3^	2.1 ± 0.1	2.3 ± 0.1	2.5 ± 0.1	2.2 ± 0.1	1.8 ± 0.3	1.5 ± 0.1	1.7 ± 0.3	1.7 ± 0.1
1,4-Xyl*p*^3^	9.8 ± 3.6	8.4 ± 1.4	11.9 ± 1.6	9.5 ± 3.0	12.3 ± 0.7	5.8 ± 0.2	4.8 ± 0.2	4.8 ± 0.6
Σ Xyl	17.0 ± 3.5	15.9 ± 1.0	19.2 ± 1.7	17.1 ± 2.8	18.5 ± 0.5	12.1 ± 0.7	11.5 ± 0.7	11.6 ± 0.4

ND, not detected; ^1^ (A1) SME 145 ± 6 Whkg^−1^, T_M_ 136 °C; (D1) SME 222 ± 10 Whkg^−1^, T_M_ 155 °C; (D2) SME 190 ± 9 Whkg^−1^, T_M_ 140 °C. ^2^ Ara, arabinose; Gal, galactose; Glc, glucose; Man, mannose; Rha, rhamnose; Xyl, xylose; *f*, furanose; *p*, pyranose. ^3^ Coeluting, calculated from the area ratio of characteristic fragment Ion peaks (*m*/*z* 117 → 1,2-Xyl*p*, m/z 118 → 1,4-Xyl*p*).

**Table 4:** Relative composition of pectic arabinan oligosaccharides liberated from chokeberry pomace powder (CPP) and extruded dietary fiber (DF) fractions ^1^ after enzymatic cleavage using endo-arabinanase (mol%, range/2, *n* = 2). IDF, insoluble dietary fiber; HMW-SDF, high molecular weight dietary fiber.

	IDF				HMW-SDF			
Compound	CPP	A1	D1	D2	CPP	A1	D1	D2
**A-2a**	81.2 ± 0.1	80.4 ± 0.1	79.8 ± 0.5	80.3 ± 0.1	84.9 ± 0.9	84.9 ± 0.2	84.4 ± 0.1	84.2 ± 0.3
**A-4a**	5.1 ± 0.1	7.2 ± 0.2	8.6 ± 0.7	8.4 ± 0.2	5.4 ± 0.4	5.2 ± 0.1	6.0 ± 0.1	5.6 ± 0.1
**A-4b**	0.9 ± 0.1	1.0 ± 0.1	<LOQ	<LOQ	1.1 ± 0.1	0.9 ± 0.2	1.1 ± 0.2	1.0 ± 0.1
**A-5b**	9.1 ± 0.1	8.8 ± 0.1	8.3 ± 0.2	8.4 ± 0.3	7.6 ± 0.1	7.9 ± 0.2	7.0 ± 0.3	7.9 ± 0.1
**A-5c**	2.4 ± 0.1	<LOQ	<LOQ	<LOQ	<LOQ	<LOQ	<LOQ	<LOQ
**A-6a**	0.6 ± 0.1	1.1 ± 0.1	1.6 ± 0.2	1.4 ± 0.1	0.4 ± 0.4	0.6 ± 0.1	0.7 ± 0.1	0.6 ± 0.1
**A-7b**	0.7 ± 0.1	1.4 ± 0.1	1.8 ± 0.2	1.6 ± 0.1	0.6 ± 0.1	0.6 ± 0.1	0.8 ± 0.1	0.6 ± 0.1

<LOQ, below limit of quantification. ^1^ (A1) SME 145 ± 6 Whkg^−1^, T_M_ 136 °C; (D1) SME 222 ± 10 Whkg^−1^, T_M_ 155 °C; (D2) SME 190 ± 9 Whkg^−1^, T_M_ 140 °C.

**Table 5:** Glucose, fructose, and sucrose contents of chokeberry pomace powder (CPP) before and after extrusion processing (g/100 g dm, mean ± SD, *n* = 2) at water contents of 13% (A1, B1, and D1) and 23% (D2), respectively. SME, specific mechanical energy (Whkg^−1^); T_M_, material temperature (°C).

Sample	SME	T_M_	Glucose	Fructose	Sucrose
CPP	---	---	5.2 ± 0.2 ^A^	4.5 ± 0.2 ^A^	0.4 ± 0.2 ^A^
A1	145 ± 6	136	4.3 ± 0.0 ^B^	3.9 ± 0.1 ^B^	0.5 ± 0.4 ^A^
B1	174 ± 4	146	4.2 ± 0.2 ^B^	3.3 ± 0.2 ^C^	0.4 ± 0.2 ^A^
D1	222 ± 10	155	3.9 ± 0.0 ^B^	3.7 ± 0.1 ^BC^	0.3 ± 0.3 ^A^
D2	190 ± 9	140	4.4 ± 0.2 ^B^	3.9 ± 0.1 ^B^	0.5 ± 0.5 ^A^

Means with different superscript capital letters within the same column differ significantly (*p* < 0.05).

**Table 6:** Anthocyanins contents of chokeberry pomace powder (CPP) and samples after extrusion processing (g/100 g dm, mean ± SD, *n* = 2) at water contents of 13% (A1, B1, and D1) and 23% (D2), respectively. SME, specific mechanical energy (Whkg^−1^); T_M_, material temperature (°C); Cy-3-gal, cyanidin-3O-galactoside; Cy-3-glu, cyanidin-3O-glucoside; Cy-3-ara, cyanidin-3O-arabinoside; Cy-3-xyl, cyanidin-3O-xyloside; Cy, cyanidin aglycon; M, monomers.

Sample	SME	T_M_	Cy-3-gal	Cy-3-glu	Cy-3-ara	Cy-3-xyl	Cy	Anthocyanins	M
CPP	---	---	1.15 ± 0.06 ^A^	0.063 ± 0.010 ^A^	0.51 ± 0.03 ^A^	0.079 ± 0.013 ^A^	<LOQ	1.80 ± 0.09 ^A^	85 ± 3 ^A^
A1	145 ± 6	136	0.38 ± 0.13 ^B^	0.021 ± 0.005 ^B^	0.17 ± 0.04 ^B^	0.025 ± 0.006 ^B^	0.010 ± 0.002 ^A^	0.61 ± 0.17 ^B^	73 ± 6 ^AB^
B1	174 ± 4	146	0.27 ± 0.02 ^B^	0.015 ± 0.002 ^B^	0.11 ± 0.01 ^BC^	0.016 ± 0.002 ^B^	0.014 ± 0.003 ^A^	0.43 ± 0.04 ^B^	68 ± 5 ^AB^
D1	222 ± 10	155	0.15 ± 0.04 ^B^	0.009 ± 0.003 ^B^	0.06 ± 0.02 ^C^	0.008 ± 0.003 ^B^	0.013 ± 0.002 ^A^	0.24 ± 0.06 ^B^	61 ± 9 ^B^
D2	190 ± 9	140	0.37 ± 0.07 ^B^	0.020 ± 0.004 ^B^	0.15 ± 0.03 ^BC^	0.022 ± 0.004 ^B^	0.013 ± 0.003 ^A^	0.58 ± 0.11 ^B^	73 ± 5 ^AB^

Means with different superscript capital letters within the same column differ significantly (*p* < 0.05).

**Table 7:** Phenolic acids contents of chokeberry pomace powder (CPP) and samples after extrusion processing (g/100 g dm, mean ± SD, *n* = 2) at water contents of 13% (A1, B1, and D1) and 23% (D2), respectively. SME, specific mechanical energy (Whkg^−1^); T_M_, material temperature (°C); 5 CQA, 5-caffeoylquinic acids acid; 4-CQA, 4-caffeoylquinic acids acid; 3-CQA, 3-caffeoylquinic acids acid.

Sample	SME	T_M_	5-CQA	4-CQA	3-CQA	Phenolic Acids
CPP	---	---	0.165 ± 0.019 ^A^	0.009 ± 0.001 ^A^	0.136 ± 0.013 ^A^	0.306 ± 0.027 ^A^
A1	145 ± 6	136	0.156 ± 0.000 ^A^	0.010 ± 0.000 ^AC^	0.126 ± 0.003 ^A^	0.292 ± 0.003 ^A^
B1	174 ± 4	146	0.159 ± 0.009 ^A^	0.012 ± 0.001 ^BC^	0.126 ± 0.005 ^A^	0.297 ± 0.014 ^A^
D1	222 ± 10	155	0.156 ± 0.006 ^A^	0.016 ± 0.001 ^D^	0.119 ± 0.002 ^A^	0.290 ± 0.008 ^A^
D2	190 ± 9	140	0.154 ± 0.004 ^A^	0.010 ± 0.001 ^AB^	0.125 ± 0.001 ^A^	0.288 ± 0.004 ^A^

Means with different superscript capital letters within the same column differ significantly (*p* < 0.05).

**Table 8:** Polyphenols contents of chokeberry pomace powder (CPP) and samples after extrusion processing (g/100 g dm, mean ± SD, *n* = 2) at water contents of 13% (A1, B1, and D1) and 23% (D2), respectively. SME, specific mechanical energy (Whkg^−1^); T_M_, material temperature (°C); TPP, total polyphenols contents analyzed by the Folin–Ciocalteu test; TPP-HPLC, total polyphenols contents calculated as the sum of monomeric anthocyanins, phenolic acids, and flavonols.

Sample	SME	T_M_	TPP	TPP-HPLC
CPP	---	---	5.5 ± 0.2 ^A^	2.29 ± 0.12 ^A^
A1	145 ± 6	136	6.2 ± 0.5 ^AB^	1.10 ± 0.18 ^B^
B1	174 ± 4	146	6.7 ± 0.6 ^AB^	0.92 ± 0.02 ^B^
D1	222 ± 10	155	7.3 ± 0.1 ^B^	0.71 ± 0.05 ^B^
D2	190 ± 9	140	6.4 ± 0.1 ^AB^	1.05 ± 0.10 ^B^

Means with different superscript capital letters within the same column differ significantly (*p* < 0.05).

**Table 9:** Techno-functional and sensory properties of chokeberry pomace powder (CPP) and samples after extrusion processing at water contents of 13% (A1, B1, and D1) and 23% (D2), respectively. SME, specific mechanical energy (Whkg^−1^); T_M_, material temperature (°C); WSI, water solubility index; WAI, water absorption index; SEI, sectional expansion index; LEI, longitudinal expansion index; H, hardness (Nm^−2^); C, color (L* lightness, a* red/green value, and b* blue/yellow value).

Sample	SME	T_M_	WSI	WAI	SEI	LEI	H	C		
								L*	a*	b*
CPP	---	---	22.3 ± 0.9 ^A^	2.9 ± 0.1 ^A^	--	--	--	29.8 ± 0.6 ^A^	21.1 ± 0.3 ^A^	5.1 ± 0.1 ^A^
A1	145 ± 6	136	23.8 ± 1.0 ^AB^	2.1 ± 0.0 ^B^	1.95 ± 0.13 ^A^	1.13 ± 0.06 ^A^	17 ± 6 ^AB^	21.7 ± 0.2 ^B^	16.9 ± 0.1 ^BC^	1.9 ± 0.0 ^BC^
A2	122 ± 5	123	24.4 ± 0.3 ^BC^	2.0 ± 0.0 ^BC^	1.52 ± 0.11 ^B^	1.15 ± 0.06 ^A^	23 ± 8 ^A^	21.1 ± 0.0 ^B^	15.3 ± 0.3 ^D^	1.8 ± 0.0 ^BCD^
B1	174 ± 4	146	25.5 ± 0.4 ^CD^	2.1 ± 0.0 ^B^	1.05 ± 0.05 ^CD^	1.57 ± 0.21 ^B^	9 ± 3 ^B^	22.1 ± 0.2 ^B^	17.2 ± 0.3 ^B^	1.9 ± 0.0 ^BCD^
B2	122 ± 5	134	25.2 ± 0.1 ^BCD^	1.6 ± 0.0 ^D^	1.49 ± 0.11 ^B^	1.47 ± 0.08 ^B^	10 ± 2 ^B^	22.0 ± 0.4 ^B^	16.6 ± 0.3 ^BCE^	2.1 ± 0.1 ^B^
C1	195 ± 10	152	26.1 ± 0.2 ^D^	1.6 ± 0.0 ^D^	0.85 ± 0.07 ^E^	2.00 ± 0.19 ^C^	8 ± 7 ^B^	21.6 ± 0.8 ^B^	16.7 ± 0.4 ^BCE^	1.7 ± 0.1 ^DE^
C2	180 ± 7	139	25.5 ± 0.2 ^CD^	2.0 ± 0.1 ^BC^	1.20 ± 0.05 ^C^	1.53 ± 0.17 ^B^	9 ± 5 ^B^	21.7 ± 0.3 ^B^	16.2 ± 0.3 ^CE^	1.7 ± 0.1 ^CDE^
D1	222 ± 10	155	26.4 ± 0.7 ^D^	2.0 ± 0.1 ^BC^	0.85 ± 0.05 ^E^	NA	8 ± 2 ^B^	23.5 ± 0.5 ^C^	17.4 ± 0.2 ^B^	1.7 ± 0.0 ^CDE^
D2	190 ± 9	140	25.4 ± 0.7 ^CD^	1.9 ± 0.0 ^C^	1.00 ± 0.12 ^DE^	1.83 ± 0.09 ^C^	13 ± 4 ^AB^	22.3 ± 0.2 ^BC^	16.0 ± 0.3 ^DE^	1.5 ± 0.1 ^E^

NA not analyzed, due to instabilities in the die. Means with different superscript capital letters within the same column differ significantly (*p* < 0.05).

## Data Availability

Data available on request.
